# Transcriptomic evidence for modulation of host inflammatory responses during febrile *Plasmodium falciparum* malaria

**DOI:** 10.1038/srep31291

**Published:** 2016-08-10

**Authors:** Tuan M. Tran, Marcus B. Jones, Aissata Ongoiba, Else M. Bijker, Remko Schats, Pratap Venepally, Jeff Skinner, Safiatou Doumbo, Edwin Quinten, Leo G. Visser, Elizabeth Whalen, Scott Presnell, Elise M. O’Connell, Kassoum Kayentao, Ogobara K. Doumbo, Damien Chaussabel, Hernan Lorenzi, Thomas B. Nutman, Tom H. M. Ottenhoff, Mariëlle C. Haks, Boubacar Traore, Ewen F. Kirkness, Robert W. Sauerwein, Peter D. Crompton

**Affiliations:** 1 Laboratory of Immunogenetics, National Institute of Allergy and Infectious Diseases, National Institutes of Health, Rockville, MD, USA; 2 Division of Infectious Diseases, Department of Medicine, Indiana University School of Medicine, Indianapolis, IN, USA; 3 Genomic Medicine Group, J. Craig Venter Institute, Rockville, Maryland, USA; 4 Mali International Center of Excellence in Research, University of Sciences, Technique and Technology of Bamako, Bamako, Mali; 5 Department of Medical Microbiology, Radboud University Medical Center, Nijmegen, The Netherlands; 6 Department of Infectious Diseases, Leiden University Medical Center, Leiden, The Netherlands; 7 Systems Immunology Division, Benaroya Research Institute, Seattle, WA, USA; 8 Laboratory of Parasitic Diseases, National Institute of Allergy and Infectious Diseases, National Institutes of Health, Bethesda, MD, USA; 9 Sidra Medical and Research Center, Doha, Qatar; 10 Infectious Diseases Group, J. Craig Venter Institute, Bethesda, Maryland, USA

## Abstract

Identifying molecular predictors and mechanisms of malaria disease is important for understanding how *Plasmodium falciparum* malaria is controlled. Transcriptomic studies in humans have so far been limited to retrospective analysis of blood samples from clinical cases. In this prospective, proof-of-principle study, we compared whole-blood RNA-seq profiles at pre-and post-infection time points from Malian adults who were either asymptomatic (n = 5) or febrile (n = 3) during their first seasonal PCR-positive *P. falciparum* infection with those from malaria-naïve Dutch adults after a single controlled human malaria infection (n = 5). Our data show a graded activation of pathways downstream of pro-inflammatory cytokines, with the highest activation in malaria-naïve Dutch individuals and significantly reduced activation in malaria-experienced Malians. Newly febrile and asymptomatic infections in Malians were statistically indistinguishable except for genes activated by pro-inflammatory cytokines. The combined data provide a molecular basis for the development of a pyrogenic threshold as individuals acquire immunity to clinical malaria.

Malaria is a significant public health burden caused by parasites of the genus *Plasmodium*, afflicting ~200 million people globally per annum^1^. Of the five species known to cause disease in humans, *Plasmodium falciparum* is responsible for the majority of malaria-related mortality. *Plasmodium* infection in the host liver is an asymptomatic process but quickly progresses to the blood stage, during which the merozoite form of the parasite invades and replicates within erythrocytes. In non-immune individuals, it is the cyclical rupture of infected erythrocytes and release of daughter merozoites during blood-stage infection that coincides with the paroxysms of fever and chills typical of clinical malaria.

Although the role of antibodies in controlling both parasite density and the symptoms of malaria is well established^2^,^3^, less is known about the contribution of cellular immune responses to host resistance to malaria symptoms during blood-stage infection^4^. We previously reported that recent malaria exposure can dampen subsequent *P. falciparum*-induced inflammation via a regulatory state mediated in part by the anti-inflammatory cytokine IL-10 produced by CD4+CD25+Foxp3- T cells that also produce IFNγ and TNF^5^. This is consistent with other studies implicating IL-10 production by polyfunctional CD4+ T cells in the modulation of malaria severity^6^,^7^. A recent report also demonstrated that tolerance to malaria disease in children with a history of heavy malaria exposure is associated with the loss and dysfunction of Vδ2+γδ T cells, which normally produce pro-inflammatory cytokines in response to infection^8^. These studies have provided valuable insight on how tolerance to malarial disease may be conferred, primarily by measuring host modulation of inflammation *in vitro* using parasite-stimulated peripheral blood mononuclear cells obtained from individuals with varying degrees of prior malaria exposure. *Ex vivo* transcriptional profiling of peripheral blood cells has provided broader and more direct insight into cellular immune responses to natural *Plasmodium* blood-stage infections in malaria-experienced individuals^5^,^9^,^10^,^11^,^12^,^13^ and during controlled human malaria infections (CHMI)^10^,^14^. The over-arching theme of these transcriptomic studies is that interferon- and toll-like-receptor-mediated inflammatory signaling and antigen presentation pathways predominate during blood-stage infections, with significant T-cell activation occurring in malaria-experienced individuals during acute malaria. However, these studies were limited to the analysis of individuals who were susceptible to clinical malaria and were not designed to prospectively identify molecular predictors and mechanisms of clinical immunity to *P. falciparum*.

We recently conducted a prospective cohort study of malaria-experienced individuals in Kalifabougou, Mali during which we collected blood samples by finger prick every 2 weeks and determined by PCR when each individual’s first *P. falciparum* infection of the malaria season occurred^15^, thereby allowing us to compare changes in immune responses before and during incident infections that did or did not progress to febrile malaria. Here, in a proof-of-principle study, we performed whole-blood RNA-seq of fingerprick blood samples collected from Malian adults who were either asymptomatic or febrile at the time of their first *P. falciparum* infection of the malaria season and compared these gene-expression profiles to those of previously malaria-naïve adults who were infected with *P. falciparum* in a CHMI study^16^. We provide evidence for disease tolerance to malaria at the transcriptome level, demonstrating that malaria-experienced individuals dampen *P. falciparum*-induced interferon and inflammatory responses during acute infection despite having higher parasite densities than naïve individuals.

## Results

### Study participant characteristics

**Figure 1:**
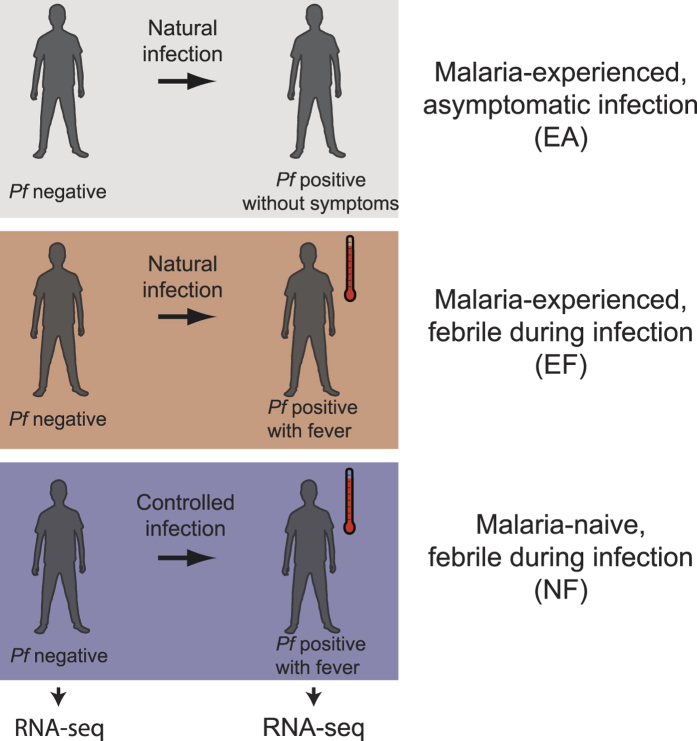
Study design and classes. RNA-seq was performed on whole blood collected prior to infection and at the first PCR-detectable *P. falciparum (Pf*) blood-stage infection in a natural malaria infection study of malaria-experienced individuals or during a controlled human malaria infection study of naïve individuals as described in the Methods.

Thirteen subjects, each with paired samples collected at their *P. falciparum* PCR-negative, uninfected baseline and during their incident, *P. falciparum* PCR-detectable blood-stage infection, were included in the group RNA-seq analyses (Fig. 1; Table 1). Eight subjects were from the Malian study, of whom 3 were febrile with uncomplicated malaria at incident infection (malaria-experienced, febrile; EF) and 5 were asymptomatic at incident infection (malaria-experienced, asymptomatic; EA). Of note, 2 individuals in the EA class progressed to febrile malaria within 5 days of initial PCR-detectable blood-stage infection, whereas the other 3 individuals carried their infections without symptoms throughout the rest of the 6-month malaria season. However, because we detected no differentially expressed genes (DEGs) between individuals in the EA class who progressed to fever and individuals in the EA class who remained asymptomatic, all 5 subjects in the EA class were analyzed as a single group. Five subjects were previously malaria-naïve individuals from the CHMI study who experienced uncomplicated febrile malaria after a single CHMI (malaria-naïve, febrile; NF). NF subjects were all women, and EF subjects were slightly younger (mean age 16.0 years; range 13.5–18.3) than subjects in classes EA (19.7 years; 15.3–23.3) and NF (20.6 years; 19.0–22.0) (Table 1). Haemoglobin levels at uninfected baseline were comparable between classes. At the incident infection, axillary temperatures for classes NF (mean 38.5 °C; range 37.7–39.4) and EF (38.5 °C; 37.7–39.4) were similar, and both were higher than class EA (36.5 °C; 36.1–37.2). Parasite densities at incident infection were lowest in class NF and highest in class EF (Table 1). All Malian individuals had a normal haemoglobin genotype AA (HbAA) except for one HbAC individual in the EF class. At the uninfected baseline, no Malian individuals were infected with *Schistosoma mansoni* by the Kato-Katz technique or with *Necator americanus, Ancylostoma duodenale, Trichuris trichiura, Ascaris lumbricoides*, or *Strongyloides stercoralis* by real-time PCR of stool genomic DNA (gDNA). Among the 6 Malians for whom urine samples were available, none were positive for *Schistosoma haematobium* by microscopy. No Malian individuals were infected by the bloodborne filarial nematode *Mansonella perstans* by PCR of blood gDNA at either the uninfected baseline or the first *P. falciparum* infection of the malaria season.

### Generation of RNA-seq data

The average number of mapped reads per sample was 71 million paired-end reads with a range of 24 to 130 million paired-end reads (Supplementary Data S1). Using a human exome size of ~50 Mb^17^ and the number of ~100 bp reads that mapped to the human genome per sample, the estimated range of coverage among our samples was 14X to 81X (Supplementary Data S1).

### Correlation of genome-wide expression across samples

Genome-wide expression of whole blood was performed using RNA-seq for paired uninfected and infected samples for all individuals (26 samples total). Hierarchical clustering of the transcriptome correlation matrix of all samples, after removal of site-specific batch effects, segregated individuals into 2 major clusters defined by *P. falciparum* infection status, regardless of whether the top 50% or the top 10% most variably expressed genes were used (Supplementary Fig. S1). Interestingly, within the uninfected cluster, individuals tended to cluster by class.

### Differential gene expression during incident *P. falciparum* infection

**Figure 2:**
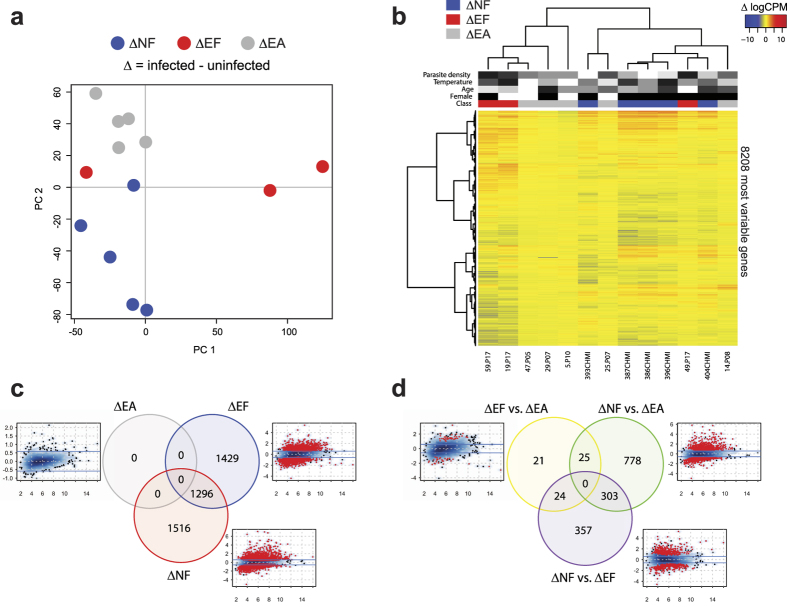
Whole-blood transcriptomic analysis of paired uninfected and infected samples demonstrates greater gene expression changes in NF than EF individuals during febrile malaria. Clinical classes were defined as in Fig. 1, and characteristics of subjects used in this analysis are in Table 1. (**a**) Principal component analysis of the paired RNA-seq samples (infected – uninfected baseline samples for each subject) using the top 50% most variably expressed genes across all paired samples after normalization of raw data (n = 13 subjects). (**b**) An unsupervised clustering heat map (Spearman correlation with Ward’s linkage) of the top 50% most variably expressed genes (8208 genes). Red intensity indicates increased expression with infection, and blue intensity indicates decreased expression with infection. Clinical classes are denoted as blue (NF), red (EF), and gray (EA). Female gender is denoted as black. Grayscale intensities represent relative rankings for age, temperature, and parasite density. Differential gene expression analysis for paired infection vs. uninfected baseline comparisons within each clinical class (**c**) and between each class (**d**) using contrasts described in the Methods. Venn diagrams show the number of differentially expressed genes (DEGs) using an absolute log_2_ fold-change (logFC) of >0.585 (1.5-fold in linear space) and a false discovery rate (FDR) <0.05. For the smear density plots, the average expression in log counts per million (x axis) is plotted against logFC (y axis), DEGs with FDR <0.05 are visualized as red points, and the fold-change cut-off is represented as blue lines.

To determine the differences in gene transcription induced by incident *P. falciparum* infection between malaria-naïve and malaria-experienced individuals with and without disease, we employed RNA-seq analysis of uninfected/infected sample pairs in a multifactorial design to account for intra-individual variation and enhance statistical power^18^ (Supplementary Data S2–S7). The majority of malaria-experienced individuals responded to *P. falciparum* infection more similarly to each other than to malaria-naïve individuals irrespective of febrile symptoms by both principal component analysis (PCA) and hierarchical cluster analysis using the top 50% (8208) most variably expressed genes (Fig. 2a,b). Despite significantly lower parasitemia (Table 1), incident infection resulted in a comparable number of transcriptional changes within the ΔNF class (2812 DEGs using an absolute fold-change threshold of >1.5 and false discovery rate [FDR] <0.05) relative to the ΔEF class (2725 DEGs), with 1296 DEGs shared between the two comparisons (Fig. 2c). At these fold-change and significance thresholds, no DEGs were observed for ΔEA (Fig. 2c). The ΔNF vs. ΔEA between-class comparison had the largest number of DEGs (1106 genes), of which 303 overlapped with the ΔNF vs. ΔEF comparison (Fig. 2d; Supplementary Data S5 and S6). The between-class ΔEF vs. ΔEA comparison revealed only 70 DEGs using our pre-set thresholds (Fig. 2d; Supplementary Data S7), despite the apparent clinical differences and large differences in the number of within-class DEGs for these two classes.

### Biological pathways affected during acute *P. falciparum* infection

**Figure 3:**
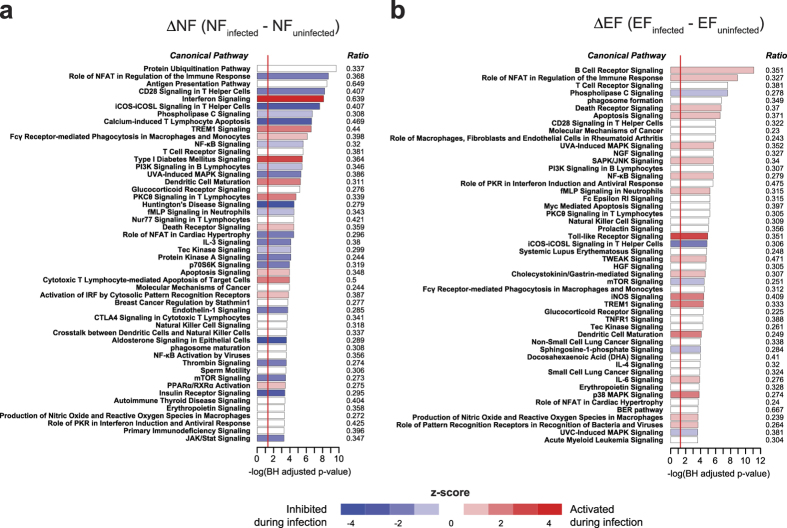
Molecular signatures of febrile malaria in naïve and malaria-experienced individuals differ in intensity and quality by pathways analysis. Canonical pathways analysis using DEGs with FDR <0.05 (no fold-change cut-off) for the ΔNF (**a**) and ΔEF (**b**) classes.

To identify the biological pathways associated with *P. falciparum* infection in naïve and malaria-experienced individuals, we determined the overlap between the DEGs determined within each of the ΔNF and ΔEF classes (FDR <0.05, no fold change filter) and Ingenuity canonical pathways by Fisher’s exact test. Using a cut-off of *P *< 0.05 (adjusted for multiple comparisons using the Benjamini-Hochberg method), acute febrile malaria significantly affected 196 pathways in the ΔNF class and 198 pathways in the ΔEF class (Supplementary Data S8 and S9), with the most significantly activated pathway being interferon signalling in the ΔNF class (Fig. 3a) and B-cell receptor signalling in the ΔEF class (Fig. 3b). Although several significantly overrepresented immune and inflammatory pathways appear in both the ΔNF and ΔEF comparisons, such as NFκB signalling and immune-related apoptosis, there tended to be higher ranking of adaptive immunity pathways (i.e. B and T cell-related signalling) in the ΔEF class relative to the ΔNF class (Fig. 3a,b).

### Predicted upstream regulators induced by acute *P. falciparum* infection

**Figure 4:**
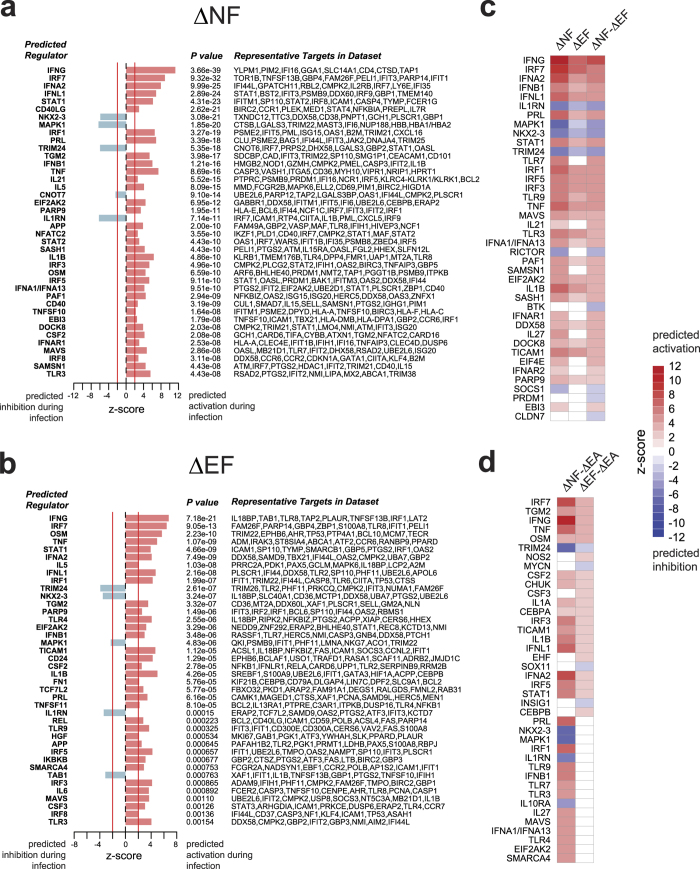
Upstream regulator analysis reveals graded activation of interferon-mediated inflammation. Upstream regulator analysis using the DEGs with FDR <0.05 (no fold-change cut-off) for the ΔNF (**a**) and ΔEF (**b**) classes. A positive z-score predicts activation of the indicated gene based on the expression patterns of downstream genes, whereas a negative z-score predicts inhibition. (**c**) Z-score data from (**a**,**b**) in heatmap format is shown with the addition of z-scores from the between group (ΔNF-ΔEF) analysis. Rows are sorted by descending Z-scores in the third column. (**d**) Z–scores in heatmap format for between-class comparisons of ΔNF and ΔEF with ΔEA using DEGS with FDR < 0.10 (no fold-change cut-off). Rows are sorted by descending Z-scores in the second column. Only the top 40 predicted regulators with an absolute z-score >2 and *P* value <0.01 are shown.

To determine which molecules might be important in the regulation of malaria immunity, we applied DEGs with a FDR <0.05 (no fold change filter) from each of the 6 contrasts outlined in the Methods (Supplementary Data S2–S7) to Ingenuity’s upstream regulator analysis to determine genes that are predicted to be important regulators during *P. falciparum* infection. Using an absolute z-score of >2 and *P* value <0.01 as the cut-off for significance, both Type I/II interferon, innate immunity, and pro-inflammatory genes were predicted to be highly activated in both the ΔNF and ΔEF classes, with IFNG and IRF7 having the strongest activation scores (Fig. 4a,b). Overall, genes related to innate immunity and inflammation were predicted to be activated to a greater degree in the ΔNF compared to the ΔEF class (Fig. 4c). IL1RN, MAPK1, TRIM24 and NKX2-3 were inhibited during infection in both classes, with significantly more inhibition in the ΔNF class relative to the ΔEF class (Fig. 4c). Although no DEGs were observed for the asymptomatically infected ΔEA class even at more relaxed cut-offs, we were still able to assess the relative differences in response to infection between this class and the ΔNF and ΔEF classes using the *between*-class contrasts. During acute infection, 98 genes were predicted to be differentially regulated between the ΔNF and ΔEA classes, with significantly stronger activation of pro-inflammatory (e.g. TNF, IL1B, RELA, NFKB1A) and interferon-related genes (IFNG, IRF7, IFNA2, IFNL1) in the ΔNF class (Fig. 4d; only top 40 regulators shown). The two malaria-experienced classes ΔEF and ΔEA differed in predicted regulation for 24 genes, 10 of which were inflammatory genes predicted to be activated in the ΔEF class relative to the ΔEA class, when DEGs with a FDR threshold of <0.10 was applied (Fig. 4d). Interestingly, among the genes predicted to be differentially activated in the ΔEF class relative to the ΔEA class are those known to encode for the endogenous pyrogens TNF, IFN-*γ* and IL-1*β*.

### Changes in modular transcriptional repertoire during acute *P. falciparum* infection

**Figure 5:**
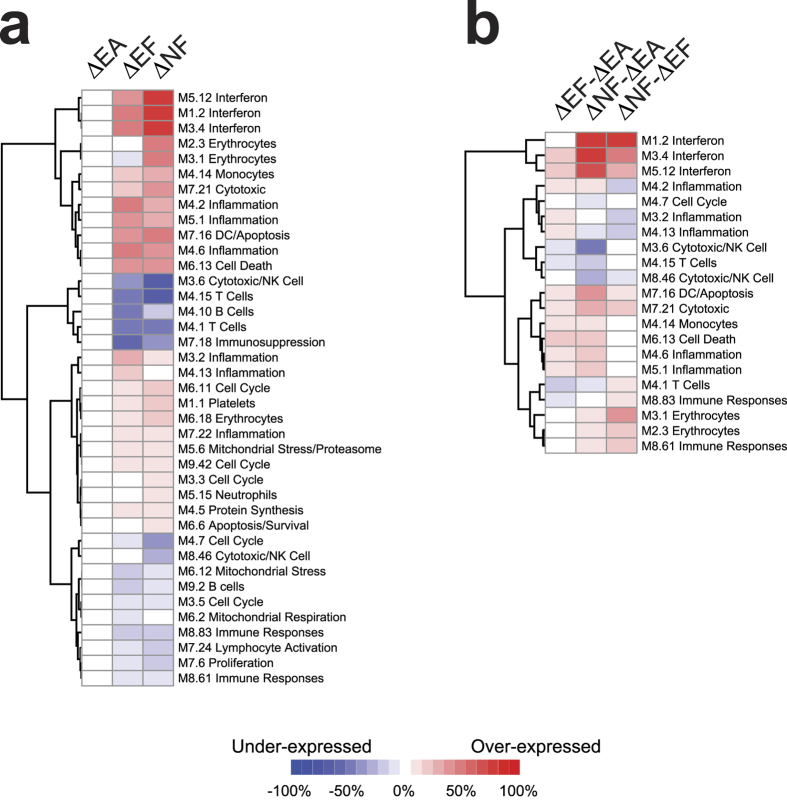
Modular transcriptional repertoire analysis shows differential induction of functional and cell subset modules in naïve and malaria-experienced individuals during febrile malaria. Modular transcriptional repertoire analyses using DEGs with FDR <0.05 (no fold-change cut-off) for within class (**a**) and with FDR <0.10 (no fold-change cut-off) for between class (**b**) comparisons. Only modules with differences in at least one comparison are shown. Rows are clustered by Euclidean distance with Ward’s linkage.

We used modular transcriptional repertoire analysis^19^ to determine the overall expression of DEGs that overlapped with modules of co-dependent gene sets known to be associated with specific cellular functions and cell subsets within each class (Fig. 5a) or between classes (Fig. 5b) during acute *P. falciparum* infection. Consistent with pathways and upstream regulator analyses, the ΔNF class had greater overexpression of interferon modules (M1.2, M3.4, M5.12) than the ΔEF and ΔEA classes (Fig. 5a,b). Differences in inflammation between the ΔNF and ΔEF classes were less consistent, with comparable overexpression in some inflammation modules (M4.6, M5.1, and M7.22) and decreased overexpression in ΔNF in others (M3.2, M4.2, M4.13; Fig. 5a,b). For the cell subset repertoires, overexpression of the monocyte module (M4.14) and underexpression of the B and T cell modules (M4.1, M4.10, M4.15, and M9.2) were seen during acute infection in both classes, in keeping with the peripheral blood monocytosis and lymphopenia commonly observed during acute malaria episodes in both semi-immune and naïve individuals during natural infection^20^ and CHMI^21^, respectively. Of note, acute malaria induced expression of genes in the erythrocyte (M2.3, M3.1, M6.18) and platelet (M1.1) modules in the ΔNF class to a greater extent than ΔEF class, (Fig. 5a), which could be due to reticulocytosis and increases in immature platelet fraction during CHMI^22^.

## Discussion

Here, we used RNA-seq to evaluate changes from pre-infection baseline in whole-blood transcription profiles during both febrile and asymptomatic natural *P. falciparum* infections in malaria-experienced Malian adults and compared these values to transcriptional changes in malaria-naïve Dutch adults who experienced fever after CHMI. We took advantage of the prospective design of a time-to-infection study in Kalifabougou, Mali and a CHMI study and used paired-sample analysis in order to eliminate baseline noise due to inter-individual variation.

Using pathways, upstream regulator and modular transcriptional analysis, we show that robust transcriptional changes during febrile malaria translated into striking quantitative and qualitative differences in the immunological response between naïve and malaria-experienced individuals, with naïve individuals having a dominant interferon-mediated, pro-inflammatory response likely driven by dendritic cells and monocytes as suggested by the modular transcriptional analysis. Despite having 300X higher parasite densities at the time of diagnosis, malaria-experienced individuals exhibited a dampened interferon-driven inflammatory response relative to naïve individuals during febrile infection, suggesting disease tolerance, but also enhanced B-cell receptor signalling not evident in naïve individuals, suggesting more robust adaptive immunity. The shorter time-to-diagnosis within the NF class due to daily monitoring for the CHMI study is unlikely to account for this difference, as it seems unlikely that a malaria-naïve individual would become more tolerant of symptoms as parasite densities increase to levels comparable to those in the EF class. Likewise, the asymptomatic EA class, which presented to clinic with 100X higher parasite densities than the NF class, provides evidence of variable disease tolerance in malaria-experienced individuals, with some individuals progressing to febrile malaria within 5 days of incident blood-stage infection, whereas the others carried their infections through the rest of the malaria season without symptoms.

Our data suggest a graded activation of pathways downstream of the pro-inflammatory cytokines IFNγ, TNF, and IL-1β that is associated with prior malaria-exposure, with naïve individuals having the greatest activation and malaria-experienced asymptomatic individuals having the least. This stands in contrast with a study by Ockenhouse *et al*. which compared transcriptomic changes in PBMCs collected during CHMI in naïve individuals at early blood-stage infection with that of malaria-experienced adults experiencing naturally acquired febrile malaria and found similar induction in pro-inflammatory cytokines IFNγ, TNF, and IL-1β between pre-symptomatic and symptomatic individuals regardless of prior malaria exposure history^10^. The likely explanation is that whereas all naïve individuals had febrile malaria (temperature >37.5) in our study, the majority of experimentally infected naïve individuals in Ockenhouse *et al*. were pre-symptomatic and therefore would not be expected to have as robust an inflammatory response as previously naïve individuals experiencing febrile malaria. A recent RNA-seq study of naïve and malaria-experienced Colombian volunteers who underwent CHMI with *P. vivax* also found no differential response in the fever-inducing inflammatory cytokines IL-1β, IL-6, IL-8 and TNF between naïve and malaria-experienced individuals at the time of diagnosis^14^. Aside from being a study on *P. vivax*, malaria-experienced subjects also had variable symptomatology at the time of diagnosis, as a majority of individuals did not exhibit fever^23^, making it difficult to make direct comparisons to our study, which separated malaria-experienced individuals into those with and without fever during incident *P. falciparum* infection.

An alternative explanation for the dampened inflammatory response observed in Malians versus Dutch individuals may be exposure to endemic helminths, which can protect individuals from febrile malaria^24^. Despite having no evidence of such infections at enrolment, modulated inflammation due to prior helminth exposure or co-infection with intestinal helminths during incident *P. falciparum* infection in the Malian individuals remains a possibility. Although we cannot completely exclude differences in ethnic or genetic background as potential confounders, none of the Malian individuals carried the malaria-protective sickle cell trait^25^, and none identified themselves as being from the Fulani ethnic group, which is known to be more protected from clinical malaria than other sympatric ethnic groups in West Africa^26^. Technical bias due to RNA-processing differences between Malian and Dutch samples is an unlikely explanation for our findings given the stable expression of non-inflammatory genes, including previously validated endogenous controls, across all samples (Supplementary Fig. S2) and the paired analysis of samples.

Despite using a paired design to enhance statistical power, small class sizes limited our ability to detect transcriptional changes during asymptomatic infections (i.e. ∆EA class), which are expected to induce more modest gene expression. Other possible reasons include the 2-week resolution for active surveillance for *P. falciparum* infection by PCR that may have missed the peak host response to initial blood-stage infection, or heterogeneity in disease tolerance within the asymptomatic group.

Although no significant changes were detected in the ∆EA comparison, 2725 DEGs were detected in the ∆EF comparison, implying robust differences in *P. falciparum*-induced gene expression between the malaria-experienced classes. Yet, direct comparison of ∆EF vs. ∆EA revealed only 70 DEGs using the same significance thresholds. Inspection of the top DEGs within the ∆EA class with an unadjusted *P* value <0.05 provides some explanation for this discrepancy (Supplementary Data S2). These top ∆EA genes are involved in similar inflammatory and innate pathways induced during febrile malaria (Supplementary Fig. S3) to an extent that might influence the between-class analyses without satisfying the threshold of FDR < 5% in the within-class ∆EA analysis. Indeed, the ∆EF and ∆EA classes were statistically indistinguishable from each other except for genes that are predicted to be regulated by TNF, IFNγ, IL-1β, which are the precise cytokines responsible for the clinical feature that differentiates the two classes: fever (Fig. 4d). Given the power limitations of our study, genes within these innate, inflammatory pathways likely reflect the largest transcriptional changes between asymptomatic and febrile *P. falciparum* infections in malaria-experienced classes. Importantly, these findings relied on the coordinated expression of known targets of transcriptional regulators and the overlap with pre-defined gene sets rather than a few isolated genes, thus reducing the likelihood of a false discovery.

The presence of a batch effect related to study-site precluded any meaningful differential gene expression (DGE) analysis between classes using only pre-infection baseline data, as the NF class and Dutch study site were synonymous. Interestingly, removal of study-site batch effects, which also removes biological variation, still resulted in higher within-class correlations than between-class correlations among pre-infection baseline transcriptomes, a finding that could be reflective of either the disparate genetic and/or environmental backgrounds between classes and/or differences in prior malaria-exposure. Although this observation requires evaluation in a larger study, the notion that pre-infection cellular signatures can predict host responses during blood-stage infection is compelling, given recent evidence that pre-perturbation cell populations can predict antibody responses induced by influenza vaccination^27^.

Dual host-pathogen RNA-seq has previously been used to investigate interactions between the human and *P. falciparum* transcriptomes using whole blood obtained during acute malaria^13^. As expected, we found a positive correlation between parasite density and *P. falciparum*-specific read counts, but the low-density infections in both the NF and EA classes precluded us from obtaining sufficient *P. falciparum* read counts in these samples for DGE analysis with the EF class. Future attempts at dual RNA-seq with low-density infections will likely require isolation of host and parasite genetic material prior to RNA collection.

In summary, we provide transcriptomic evidence of disease tolerance in human malaria, demonstrating regulated interferon-mediated inflammatory responses in malaria-experienced individuals during *P. falciparum* blood-stage infections that would otherwise induce intense inflammation in naïve individuals at much lower parasite densities. Differences in the molecular intensity of febrile malaria are accompanied by qualitative differences, with prominent activation of B-cell receptor signalling that is unique to malaria-experienced individuals. Taken together, these data provide a molecular basis for the development of a pyrogenic threshold as individuals acquire natural immunity to malaria disease. Larger studies are needed to validate these findings and to gain further insight into the cellular immune response during asymptomatic *Plasmodium* infections.

## Methods

### Ethics Approval

Written informed consent was obtained from all subjects. The Malian cohort study was approved by the Ethics Committee of the Faculty of Medicine, Pharmacy and Dentistry at the University of Sciences, Technique and Technology of Bamako and the Institutional Review Board of the National Institute of Allergy and Infectious Diseases, National Institutes of Health. The study in Mali is registered on http://www.clinicaltrials.gov (NCT01322581). The CHMI study was approved by the Central Committee for Research Involving Human Subjects of The Netherlands (NL33904.091.10) and complied with the Declaration of Helsinki and Good Clinical Practice.

### Malian Study Site, Study Population and Sample Collection

The malaria-experienced individuals for this study are part of a larger on-going prospective cohort study of naturally acquired malaria immunity^15^,^24^. Briefly, the study was conducted in Kalifabougou, Mali, where *P. falciparum* malaria transmission is intense and seasonal, occurring from June through December^15^. In May 2011, we enrolled 695 healthy children and adults, aged 3 months to 25 years, into a longitudinal observational cohort study to investigate malaria immunity in which we conducted bi-weekly active surveillance for *Plasmodium* infection and weekly active and passive surveillance for clinical malaria. Exclusion criteria included a haemoglobin level <7 g/dL, axillary temperature ≥37.5 °C, acute systemic illness, underlying chronic disease, use of antimalarial or immunosuppressive medications in the past 30 days, or pregnancy. Haemoglobin typing for HbS and HbC was performed with a D-10 instrument (Bio-Rad). Malaria episodes were pre-defined as parasitaemia of ≥2500 parasites/μL, an axillary temperature of ≥37.5 °C within 24 hours, and no other cause of fever discernible by physical exam. By finger prick we collected whole-blood for RNA (200 μl whole blood in 400 μl of Tempus solution [Applied Biosystems]), dried blood spots (DBS) on filter paper (Protein Saver 903, Whatman), cell pellets, and plasma from healthy, uninfected children and adults at enrolment (before the 6-month malaria season), during bi-weekly scheduled visits, and at their first malaria episode of the ensuing transmission season. After transport from the field site, RNA, blood pellets, and plasma were stored at −80C until analysis. Point-of-care blood smears were performed for individuals symptomatic at any clinic visit, with anti-malarials given for any *Plasmodium* positive smears per the Malian national guidelines. For each subject, the first *P. falciparum* infection of the malaria season was detected retrospectively by PCR analysis of the longitudinally collected DBS^15^, and positive samples were then quantified by qPCR^28^. First malaria episodes were determined from the clinical visit data.

Here, we focus on individuals >13 years old, which is the age when levels of *P. falciparum*-specific antibodies approach adult-like levels in this cohort^29^. From this subset, we randomly selected 1) individuals who were febrile at the time of their first PCR-detected *P. falciparum* infection (malaria-experienced, febrile; EF) and 2) individuals who were asymptomatic at the time of their first PCR-detected *P. falciparum* infection (malaria-experienced, asymptomatic; EA) for whom samples were available for RNA-seq.

### Screening for helminth co-infections in Malian subjects

Screening for helminths at uninfected baseline (enrolment) was performed as previously described^24^. Briefly, urine was assessed for *Schistosoma haematobium* eggs by microscopy after urine filtration. Presence of *Schistosoma mansoni* eggs was assessed by microscopy of stool using the Kato-Katz technique^30^. The intestinal nematodes *Necator americanus, Ancylostoma duodenale, Trichuris trichiura, Ascaris lumbricoides*, and *Strongyloides stercoralis* were detected following DNA extraction from stool^31^ by multi-parallel, real-time PCR^32^. In addition, PCR-detection of the bloodborne filarial nematode *Mansonella perstans* was also performed on gDNA extracted at both the uninfected baseline and the first *P. falciparum* infection of the malaria season with two 3-mm DBS punches using an established protocol^33^.

### Controlled human malaria infections

The malaria-naïve individuals in this study, all of whom were febrile during *P. falciparum* infection (malaria-naïve, febrile; NF) included five individuals who were recruited as infectivity controls for a previously described CHMI study (NCT01218893)^16^. Briefly, all five individuals tested negative for *P. falciparum*-specific antibodies by ELISA at enrolment, and none had travelled to a malaria-endemic area within 6 months prior to the study. In October 2011, individuals were challenged simultaneously by exposure to bites of five *Anopheles stephensi* mosquitoes infected with the NF54 *P. falciparum* strain and monitored by daily thick blood smear starting on days 5–6 post-challenge. Whole blood was collected for RNA in PAXgene blood RNA tubes (PreAnalytiX) on the day prior to challenge; days 5, 6 and 9 after challenge; the day of treatment; and day 35 after challenge. In addition to blood-smear diagnosis, parasite density was also retrospectively quantified by qPCR of blood DNA samples collected up to twice daily from day 5 until day 21 after challenge to determine the time to PCR-positivity as described previously^16^. Curative anti-malarial treatment with atovaquone/proguanil was provided immediately after patent parasitaemia was detected.

### Generation of RNA-seq data

For all Dutch samples, total RNA was extracted from whole blood using the PAXgene Blood miRNA Kit (Qiagen). RNA was converted to cDNA and amplified using Ovation RNA-Seq System V2 (Nugen), which uses single-primer isothermal amplification equivalent to 12 cycles of PCR amplification, and library preparation was performed with the TruSeq Nano Kit (Illumina) per the manufacturers’ instructions. For the Malian samples, total RNA was extracted from whole blood using the Tempus Spin RNA isolation kit (Thermo Fisher) and depleted of rRNA and globin RNA before amplification using the ScriptSeq Complete Gold Kit (Illumina). Directional RNA-seq libraries were prepared using the ScriptSeq v2 kit with barcode adapters (Epicentre), which includes a 15-cycles of PCR amplification. For both Malian and Dutch samples, sequencing of 2 × 100 bp paired-end reads was performed on a HiSeq 2000 using V3 reagents (Illumina). The Illumina sequences were trimmed of bases with a Phred quality score of less than 15 and any contaminating adapters used in the preparation of cDNA and sequencing libraries by the Trimmomatic program^34^. Only paired-end reads which survived trimming and were ≥60 bases in length were mapped to the human (GRCh37, version 17, Ensembl 72) and *Plasmodium falciparum* 3D7 (version 3 annotation, Dec 2012) genomes in parallel using TopHat2 35. Transcript abundance was determined by Cufflinks^36^. Although samples from the Malian and Dutch studies were processed and sequenced at separate times, paired samples from the same subject were always processed together. Sequencing data is available on the Gene Expression Omnibus (GEO) database under the accession numbers GSE52166 (natural infection) and GSE50957 (CHMI).

### Gene expression analysis

Gene expression analysis of RNA-seq read counts mapping to the human genome was performed using edgeR (version 3.10.2)^37^. Filtering and normalization were performed for uninfected and infected samples simultaneously prior to downstream paired analyses. After removal of genes on the Y chromosome, TMM normalization was applied, followed by removal of low-expression genes (genes with <1 count per million [CPM] in ≥10% of the samples). To verify that RNA processing differences in the Malian and Dutch samples did not produce a technical bias in genome-wide expression levels, we evaluated expression of 16 genes previously identified as being stable endogenous controls across a panel of human tissues^38^ and in human whole blood across multiple disease states^39^ and found their expression to be comparable between Malian and Dutch samples (Supplementary Fig. S2).

Expression data for the top 50% most variable genes by median absolute deviation^40^ were converted to logCPM, and unwanted variation (i.e. study site-specific batch effects) were removed using the remove BatchEffect function in the limma package prior to generation of correlation matrices, PCA plots and unsupervised hierarchical clustering heatmaps. For data visualization in the paired analysis, logCPM_infected_ − logCPM_uninfected_ (delta logCPM) values were used for each subject. We compared the effect of infection within subjects as well as the differences between classes while taking into account the pairing of samples by subject. We used the following model formula which has class as the main effect with nested interactions for subject and infection status: ~Class + Class:Subject + Class:Infection. Thus, differences in batch, sex, and age were accounted for by the subject effect, as each subject’s pre-infection baseline served as his or her own control. DGE analysis was performed by fitting counts for each gene to a negative binomial generalized log-linear model using the glmLRT function for the following *within* class comparisons, set up as contrasts:


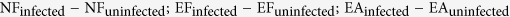


*Between*-class comparisons were also performed:


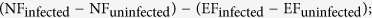



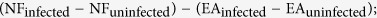



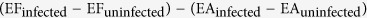


Gene symbols, log-fold change ratios, unadjusted *P* values, and false discovery rates from results of the model were imported into Ingenuity Pathway Analysis (IPA, Qiagen) to determine pathway enrichment scores and perform upstream regulator analyses and into a custom R script for modular transcriptional repertoire analysis^19^,^41^.

### Evaluation of *P. falciparum*-specific read counts

Our initial strategy of simultaneously mapping read counts to both human and *P. falciparum* reference genomes sought to determine relationships between the host and parasite transcriptomes in minimally processed finger prick whole blood samples, with the goal of comparing parasite transcriptomes in both asymptomatic and febrile infections. Such a strategy yields sufficient human but not *P. falciparum* mapped counts, presumably due to the overwhelming abundance of human transcripts in whole blood un-enriched for parasite material, with adequate library sizes for *P. falciparum* only observed at high parasite densities (>10,000 parasites/μl; Supplementary Fig. S4).

### Statistical analysis

Statistical analyses were performed in R 3.2.3 (https://cran.r-project.org) as indicated in the results or figure legends. Statistical significance was set at FDR <0.05 unless noted otherwise.

## Additional Information

**How to cite this article**: Tran, T. M. *et al*. Transcriptomic evidence for modulation of host inflammatory responses during febrile *Plasmodium falciparum* malaria. *Sci. Rep.* **6**, 31291; doi: 10.1038/srep31291 (2016).

## Supplementary Material

Supplementary Information

Supplementary Data S1

Supplementary Data S2

Supplementary Data S3

Supplementary Data S4

Supplementary Data S5

Supplementary Data S6

Supplementary Data S7

Supplementary Data S8

Supplementary Data S9

## Figures and Tables

**Table 1:** Participant Characteristics.

	EA	EF	NF	*P* value
Number	5	3	5	
% Female	40	66.7	100	NSa
Mean age in years (range)	19.7 (15.3–23.3)	16.0 (13.5–18.3)	20.6 (19.0–22.0)	NS^b^
Mean hemoglobin (g/dL) at uninfected baseline (range)	14.1 (12.1–17.6)	13.2 (12.6–13.6)	13.1 (12.4–14.3)	NS^b^
Mean temperature (°C) at first infection (range)	36.5 (36.1–37.2)	38.5 (37.7–39.4)	38.5 (37.7–39.4)	NA
Mean parasite density (parasites/μl) at first infection (range)	5100 (1000–8100)	22,000 (14,000–31,000)	57 (4.1–100)	0.0051^b^

Abbreviations: EA, malaria-experienced, asymptomatic at first infection; NF, naïve, asymptomatic at first infection; EF, malaria-experienced, febrile at first infection; NS, not significant; NA, not applicable. ^a^Fisher’s exact test. ^b^Kruskal-Wallis test.
