# Supplementary material for: Transcriptomic evidence for modulation of host inflammatory responses during febrile Plasmodium falciparum malaria
Source: Sci Rep. 2016 Aug 10;6:31291. doi: 10.1038/srep31291 (PMC4978957; doi:10.1038/srep31291)
Supplement: Supplementary Information [file srep31291-s1.doc]

**SUPPLEMENTARY INFORMATION**

**Transcriptomic evidence for modulation of host inflammatory responses during febrile *Plasmodium falciparum* malaria**

Authors:

Tuan M. Tran^1,2*^, Marcus B. Jones^3^, Aissata Ongoiba^4^, Else M. Bijker^5^, Remko Schats^6^, Pratap Venepally^3^, Jeff Skinner^1^, Safiatou Doumbo^4^, Edwin Quinten^6^, Leo Visser^6^, Elizabeth Whalen^7^, Scott Presnell^7^, Elise M. O’Connell^8^, Kassoum Kayentao^4^, Ogobara K. Doumbo^4^, Damien Chaussabel^7,9^, Hernan Lorenzi^10^, Thomas B. Nutman^8^, Tom H.M. Ottenhoff^6^, Mariëlle C. Haks^6^, Boubacar Traore^4^, Ewen F. Kirkness^3^, Robert W. Sauerwein^5^, and Peter D. Crompton^1^

Affiliations:

^1^ Laboratory of Immunogenetics and ^8^Laboratory of Parasitic Diseases, National Institute of Allergy and Infectious Diseases, National Institutes of Health, Rockville, MD, USA

^2^ Division of Infectious Diseases, Department of Medicine, Indiana University School of Medicine, Indianapolis, IN, USA

^3^ Genomic Medicine Group and ^10^Infectious Diseases Group, J. Craig Venter Institute, Rockville, Maryland

^4^ Mali International Center of Excellence in Research, University of Sciences, Technique and Technology of Bamako, Bamako, Mali

^5^ Department of Medical Microbiology, Radboud University Medical Center, Nijmegen, The Netherlands

^6^ Department of Infectious Diseases, Leiden University Medical Center, Leiden, The Netherlands

^7^ Systems Immunology Division, Benaroya Research Institute, Seattle, WA, USA

^9^ Sidra Medical and Research Center, Doha, Qatar

*Correspondence to tuantran@iu.edu

**Supplementary Fig. S1. *P. falciparum* infections drive gene expression profiles more than malaria-immune status.** Correlation matrices derived from normalized expression data using the top 50% **(a)** and top 10% **(b)** most variably expressed genes, obtained after removal of site-specific batch effects, were applied to unsupervised clustering heatmaps (Spearman correlation with Ward’s linkage). Purple shading represents the Pearson r for each pairwise comparison.

**Supplementary Fig. S2. Stable expression of previously validated endogenous controls across all samples despite differences in RNA processing between Malian and Dutch samples.** RNA-seq read counts for all 26 uninfected and infected samples were normalized using edgeR as in the Supplementary Data and displayed as counts per million (CPM). Expression levels for all 16 genes were not significantly (NS) different between Malian (EA and EF) and Dutch samples (NF) by t test or Wilcoxon test as appropriate.

**Supplementary Fig. S3. Upstream regulator analysis suggests subtle inflammatory responses during incident asymptomatic *P. falciparum* infection.** Z-scores from upstream regulator analysis using the DEGs with FDR <0.05 (no fold-change cut-off) for the ΔNF and ΔEF classes and DEGs with unadjusted *P* < 0.05 (no fold-change cut-off) for the ΔEA class. Only the top predicted regulators with an absolute z-score > 2 and *P* value < 0.01 are shown. Rows are sorted by descending Z-scores for the ΔEA column followed by the ΔEF column.**Supplementary Fig. S4. Whole-blood RNA-Seq mapped *P. falciparum* read counts are insufficient when parasite densities are less than 10,000 parasites/μl.** Library size is shown as counts per million (CPM), and parasite density was determined by qPCR as described in the Methods.
